# Outcome of Physiotherapy Treatment on a 28-Year-Old Male Diagnosed With Avascular Necrosis

**DOI:** 10.7759/cureus.48342

**Published:** 2023-11-06

**Authors:** Medhavi V Jagzape, Deepak P Jain, Deepali S Patil

**Affiliations:** 1 Department of Musculoskeletal Physiotherapy, Ravi Nair Physiotherapy College, Datta Meghe Institute of Medical Sciences, Wardha, IND

**Keywords:** hip, femoral head, avascular necrosis, case report, physiotherapy

## Abstract

Avascular necrosis (AVN) of the femoral head is a chronic condition that primarily affects patients under the age of 40. While the precise etiology of AVN remains unknown, the condition is defined by a vascular insult to the femoral head's blood supply, which can cause the femoral head to collapse and then undergo degenerative alterations. As the condition worsens, the articular surface may collapse depending on how much of the femoral head is affected. When the femoral head collapses in these people, significant pain follows, and the condition seldom regresses. The patient came to Acharya Vinoba Bhave Hospital Outpatient Department of Orthopedics with a complaint of bilateral hip pain (right > left). The patient had a history of COVID-19, for which the patient took steroids of high dosage, and later he had a complaint of bilateral hip pain that was gradual and progressive, which affected the daily living activities of the patient for which the patient was operated for the bilateral hips. Postoperatively, the patient has been given a physiotherapy call, which included isometric exercises, stretching and strengthening exercises, which have shown recovery in the patient.

## Introduction

Avascular necrosis of the femoral head (ANFH) is a common condition marked by the apoptosis of bone cells, including bone marrow, bone-forming cells, and bone-destroying cells. As a result, the bone collapses, and the cartilage above it becomes involved, flattening the head's surface and eventually leading to secondary osteoarthritis. [1]. It was estimated in the early 2000s that the general population of the United States experienced between 300,000 and 600,000 cases of osteonecrosis of the femoral head (ONFH), which is characterized by the progressive necrosis of bone cells and bone marrow [2]. Osteonecrosis of the femoral head (ONFH) typically affects the anterior and superior parts of the femoral head, but the prevalence of this condition is unknown as the early stages can be subtle [3]. After healing from the COVID-19 acute stage, the disease's long-term effects (such as chronic arthritis) could last for weeks or even months. This is when the term "Long COVID" was coined. Based on the findings from the 2003 SARS Epidemic, when it was reported that the number of cases of femoral head avascular necrosis (FHAVN) in infected patients had increased to between 23% and 28.8%, the main cause of this increase was attributed to the widespread use of corticosteroids to treat respiratory symptoms and reduce the inflammatory response (AVN) or osteonecrosis has been documented in a number of joints, including the spine, shoulders, and knees, with the hip joint being the most affected by far [4].

Several internal and external variables contribute to intramedullary microvascular lesions in steroid-induced ONFH, which is a complex clinical condition that causes the femoral head to receive insufficient blood and oxygen, promoting osteocyte death. The specific etiology and molecular pathways responsible for the beginning of the illness remain unknown [2]. The femoral head may suffer irreparable damage as the disease progresses, which has a significant impact on the patient's quality of life. As a result, research into the pathophysiology of SONFH and its clinical therapeutic benefits is crucial for prevention, diagnosis, and treatment. Even though it is now thought that SONFH is the result of numerous circumstances, the precise pathologic process that causes it remains unclear. We examine five possibilities for the pathophysiology of SONFH in this article. In addition to trauma, other nontraumatic variables that contribute to ON-FH include hormone usage, alcoholism, coagulopathy, sickle cell disease, and others [5].

The most frequent among them is glucocorticoid-related femoral head necrosis (GA-ONFH). Glucocorticoid users who use them for a long time are more likely to develop GA-ONFH [6]. Early events may include vascular injury, mechanical stresses, elevated intraosseous pressure, adipocyte dysfunction, apoptotic abnormalities, and coagulation malfunction. Nevertheless, as nutrients cannot reach the watershed regions of the femoral head, the common pathway ultimately results in bone loss [7]. Lack of blood supply: steroids can harm arteries and result in endothelial loss, arterial smooth muscle necrosis, vessel wall necrosis, and luminal blockage, which in turn lowers blood flow and results in osteonecrosis. Steroids may encourage osteoblastic and osteocytic apoptosis, impairing the osteocyte network's ability to sense mechanical forces and leading to the collapse of the femoral head [8]. The notch-signaling pathway, which is connected to the proliferation and differentiation of bone marrow mesenchymal stem cells, may be diminishing, which may be one of the mechanisms contributing to the loss of postmenopausal bone mass in osteoporosis patients [9]. Although pulse therapy for transplant rejection was not modified or did not even lower the AVN rate in two trials including renal transplant patients, the relationship between high-dose pulse or bolus steroid therapy and AVN has not been well investigated [10]. Frequently, a root cause cannot be identified. These idiopathic cases, however, might be caused by clotting irregularities or collagen mutations. According to Jones et al., 82% of the patients in their study had at least one aberrant coagulation factor [11]. Recently, physiotherapy treatments such as strengthening and non-weight-bearing ambulation with crutches after core decompression have been found to be effective in patients with avascular necrosis [12].

## Case presentation

Patient information

A 28-year-old male was brought to Acharya Vinoba Bhave Rural Hospital Ortho Outpatient Department on June 18, 2022, with a complaint of pain over both hips for a period of two months. The pain began slowly, was dull and painful, and was initially minimal. It was made worse by movement and exertion and was soothed by rest and medication. The pain progressed over two months to the extent that the patient was having difficulty in day-to-day activities like sitting in the cross-legged position. The patient complained of difficulty walking because of pain over the right hip. The patient does not have any history of fever, constitutional symptoms that may affect the well-being of the patient or swelling. There is no history of blood transfusions or alcohol intake. There is no significant family history. The patient has a history of COVID-19 for which the patient took steroids. The patient was prescribed medications for the relief of pain and was advised for an X-ray of his hip joint, for which he came to Acharya Vinoba Bhave Rural Hospital. An X-ray revealed that there were increased densities with small lytic lesions in both the femoral head and irregularities in the shape of the head. Magnetic resonance imaging of bilateral hips was conducted on June 19, 2022, which revealed early stage 2 avascular necrosis of both femoral heads, for which he underwent surgery named core decompression for both hips on June 26, 2022, and was referred for physiotherapy on June 27, 2022.

Clinical findings

According to the numerical pain rating scale, the patient's pain is rated as 5/10 at rest and 8/10 while in movement on postoperative day one. Prior to receiving physiotherapy, the patient's range of motion was limited by pain in both hips, and a resisted isometric contraction revealed that the patient's muscles were weak and painful. The patient was admitted on June 18 and underwent the first investigation, an X-ray, the following day. On June 26, 2022, the patient underwent a procedure, and on July 3, 2022, the patient was discharged from the hospital.

Diagnostic assessment

**Figure 1:**
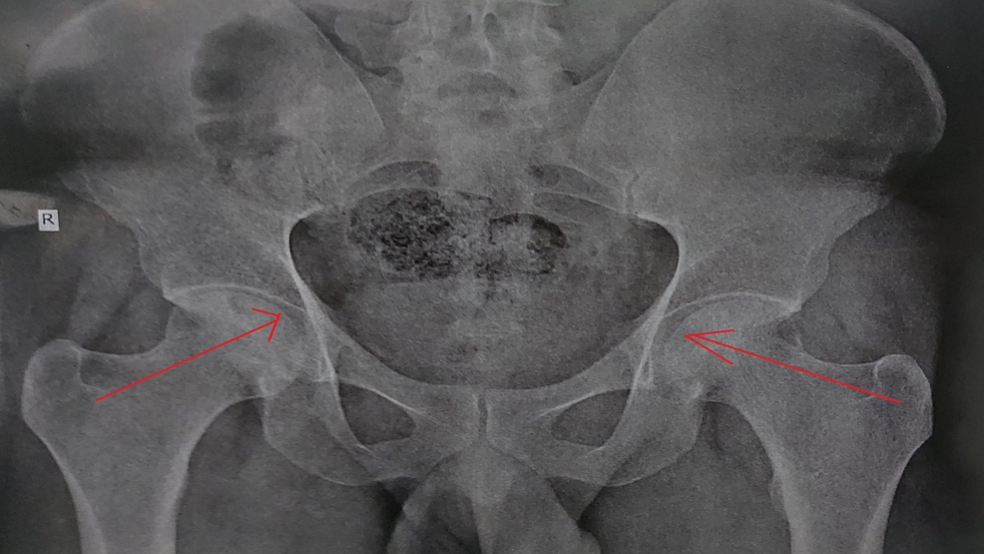
X-ray of a 28-year-old male in the anterior-posterior view shows the avascular necrosis of the bilateral head of the femur

The patient underwent several investigations such as an X-ray (Figure 1) and magnetic resonance imaging. The X-ray findings revealed avascular necrosis of the bilateral head of the femur, and the magnetic resonance imaging revealed grade avascular necrosis in both hip joints according to modified Ficat and Arlet showing more than 50% femoral head involvement on the right or less than 50% femoral head involvement on the right more than the left femoral head and neck morrow edema and maintained bilateral femoral head sphericity and cartilage. 

Therapeutic interventions

The patient underwent a surgical intervention named core decompressive surgery, and postoperative day one, a physiotherapy call was advised, and he was treated for four weeks. Table 1 describes the management from day one to week one, while Table 2 describes the diagnosis from week two to week four.

**Table 1:** Physiotherapy intervention (day 1 to week 1)

Treatment	Rationale
Static quadriceps	To initiate the action of the quadriceps muscle
Static hamstrings	To initiate the action of the hamstrings
Static gluteals	To initiate the action of the gluteal muscles
Static abdominals	To strengthen the abdominal muscles
Heel slides within the available range	To maintain the available range of motion
Upper limb strengthening with 500 gm	To increase the strength of the muscles in the upper limb
Positioning	To prevent bed sores

**Table 2:** Physiotherapy intervention (week 2 to week 4) SLR (straight leg raise)

Treatment	Rationale
Heel slides	To increase the range of motion
SLR	To strengthen the hip flexors
Pelvic bridging initiation	To maintain core stability
Sit to stand with the help of a walker	To initiate standing and balance
Bedside sitting	To initiate sitting and trunk control and weight bearing on the bilateral hips
Dynamic quads	To maintain and increase the range of knee extension

Follow-up and outcome of interventions

The patient's pain level was 3/10 during movement, the ranges (Table 3) were measured by the goniometer, and the muscle strength (Table 4) was evaluated after four weeks of intervention, whereas it was not during the baseline because of pain. These changes were observed in the patient's pain level, movement ranges, and muscle strength. The outcome indicators varied between baseline and four weeks during the intervention. The lower extremity function scale was 5% at the baseline and 32.5% after four weeks of the intervention, whereas Harris' hip score was determined to be 11 at baseline and 63 after the intervention.

**Table 3:** Post-intervention range of motion (after four weeks)

MOVEMENT	RANGES
Hip flexion	0-120 deg
Hip extension	0-20 deg
Hip abduction	0-20 deg
Knee flexion	0-120 deg
Knee extension	120-0 deg

**Table 4:** Post-intervention: manual muscle testing (after four weeks)

MOVEMENT	GRADES
Hip flexion	3
Hip extension	3
Hip abduction	3
Knee flexion	3
Knee extension	3

## Discussion

There are a number of established risk factors for avascular necrosis, including bone fractures, steroids, and alcoholism, even if the exact causes remain unknown. Numerous disorders, particularly those affecting the immune system or the musculoskeletal system, are commonly treated with steroids. In actuality, it is thought that high-dose steroids are the main nontraumatic cause of ANFH [13]. Two steps can be used to restore necrotic bone: (1) the development of osteoblasts, which produce bone tissue on the surface of defunct trabeculae, from mesenchymatous cells, and (2) cellular growth with repair tissue invasion of the skull. Post-traumatic necrosis repair tissue grows to create new bone after crossing the subcapital fracture line. Because intra-head microfractures cause mesenchymatous cells to differentiate into fibroblasts, which slow the process by producing a fibrous layer similar to that found in nonunion, they limit osteoblast growth and osteogenesis [14]. Predisposing risk factors might not be identified in some patients. Furthermore, only a very small percentage of people exposed to a particular risk factor go on to develop the disease. For instance, in two separate studies by Zalavras et al. that looked at the start of osteonecrosis after exposure to high corticosteroid doses, osteonecrosis was infrequently identified. Only three of 203 recipients of liver transplants (2%) in the first experiment experienced symptomatic hip osteonecrosis. Only six of 204 patients (3%) in the second experiment, which was similar to the first, developed osteonecrosis of the hip or knee after heart donation. This information suggests that additional genetic components are required for a patient to develop symptomatic disease, and it may be used to support the theory that osteonecrosis is a complex disease [15]. Medications like glucocorticoids make the vascular mechanisms that cause thrombotic occlusion more powerful. They exacerbate hypercoagulability and harm endothelial cells directly. Glucocorticoids subdue myofibroblastic activity, which in turn inhibits capillary growth [16]. For the treatment of painful ANFH in middle-aged adults, numerous surgical techniques have been documented. Various intertrochanteric femoral osteotomies, free or vascularized bone grafting, core decompression, and other joint-preserving procedures are currently offered. Sugioka created a novel method for treating particular forms of femoral head avascular necrosis called trans trochanteric rotational osteotomy. Physiotherapy-assisted active exercises were encouraged and continued for months. Non-weight-bearing on crutches was then advised until there was roentgenological evidence of solid union, which would indicate early recovery of the patient. In this case, the patient was non-weight bearing for four weeks, but static and in-bed exercises were started on postoperative day one, which increased the mobility and strength of the patient [17]. An investigation in the 2022 results of adapted physical therapy for postoperative avascular necrosis treated in this instance by core decompression and PRP infiltration. The patient complained of pain, limited range of motion (ROM), muscle weakness, and trouble bearing weight when they were referred to adapted physical therapy. The goal of rehabilitation was to regain the full range of motion, improve weight bearing gradually, and maintain the strength of the corresponding muscles. The patient had treatment for eight weeks, five days a week. Bedside sitting, static quadriceps strengthening exercises, and mobilization were important rehabilitative exercises. It is concluded that the modified physical therapy method utilized in this case study was successful in promoting early strength, range of motion, and functional activities in patients with post-operative avascular necrosis. In this study, the same protocol was advised, but the surgeon advised non-weight bearing for four weeks so it was not included in the physiotherapy treatment [12].

## Conclusions

Surgical intervention along with physiotherapy treatment improved the patient's range of motion of the hip as well as the knee and strength in both extremities, which made the patient independent in their activities of daily living. Initially, the patient was unable to sit gradually. After physiotherapy treatment, the patient could sit without support. Initially, the range of hip and his muscle strength was low, but now he can move his hip and perform his activities on his own and can also perform activities of daily living with minimal support.
